# No Resilience Without Partners: A Case Study on German Small and Medium-Sized Enterprises in the Context of COVID-19

**DOI:** 10.1007/s41471-022-00149-5

**Published:** 2022-12-15

**Authors:** Anna Trunk, Hendrik Birkel

**Affiliations:** Chair of Supply Chain Management, Friedrich-Alexander University Erlangen-Nuremberg, Lange Gasse 20, 90403 Nuremberg, Germany

**Keywords:** Supply chain management (SCM), Small and medium-sized enterprises (SME), COVID-19, Case study, Resilience, D23, D8, H12, L14, L22, M19, M39

## Abstract

Much research has been conducted on the effects of COVID-19 on company and supply chain resilience. However, few contributions have focused on small and medium-sized enterprises. These companies are claimed to be the drivers of economic growth but often lack access to resources and alternatives when interruptions occur, making them a bottleneck for supply chains. Using a multiple case study approach, this paper links resilience theory to the design of the relationships between eight German small and medium-sized enterprises and their suppliers and customers. It analyzes the way in which these companies combine contractual and relational investments across their supply chain flows of product, finance, and information in order to improve resilience. Company representatives were interviewed on three occasions between June 2018 and December 2020, that is, before COVID-19 and during the lockdowns. The results of the case study explain why and how companies of this type have been able to anticipate and manage the crisis. The interviews revealed that those companies that made the largest investments in the relational aspects of their partnerships while safeguarding product and financial flows through contracts performed best. In principle, contractual investments are higher in partnerships with suppliers. However, the precise combination of contractual and relational investments depends on the business model, the business philosophy of the CEO, and the allocation of power within the supply chain. These findings indicate that, when collaborating with small businesses, supply chain partners should focus on building relationships in order to create resilience in the supply chain.

## Introduction

“Resilience is becoming a buzzword” (Walker 2020). The concept of resilience was introduced in the 1960s in the context of natural ecosystems. Today, members of the public and academics also discuss it in the context of individuals and organizations (Folke 2006; Walker 2020; Wieland 2021). Since no common definition exists, Walker (2020) summarized resilience as “the ability to adapt and change, to reorganize, while coping with disturbance.” In today’s globalized world, companies are parts of supply chains (SCs), a system of various stakeholders that need to act together in order to generate a final product (Bak et al. 2020; Folke 2006; Ren et al. 2011). The COVID-19 pandemic demonstrated that the individual challenges that one partner faces have the potential to affect the system as a whole. At the same time, the coping and risk-management strategies of individual organizations are not adequate and adaptable to the complexity of an entire SC (Dubey et al. 2021; Folke 2006; Wieland and Durach 2021).

The literature on SC resilience is fragmented (for an overview, see Han et al. 2020; Davis-Sramek and Richey 2021; Dubey et al. 2020). This said, research in the context of COVID-19 has been particularly bountiful since the beginning of 2020 (Bak et al. 2020; Chowdhury et al. 2021; Ivanov and Dolgui 2021). In line with Buyl et al. (2022), SC resilience, for the purpose of this article, is defined as “the capacity of a supply chain to persist, adapt, or transform in the face of change.” (Wieland and Durach 2021, p. 316) Studies usually concentrate on specific types of SCs and situations (for an overview, see Kochan and Nowicki 2018; Wieland and Durach 2021). Chowdhury et al.’s (2021) literature review demonstrated that the focus of articles on COVID-19 has been on SCs for high-demand and healthcare products. Thus, the impact that COVID-19 has had on the resilience of companies that are active in other industries, as well as on different roles and company types within an SC, remains understudied.

The foregoing is particularly relevant to small and medium-sized enterprises (SMEs; Chowdhury et al. 2021). SMEs are seen as important partners in SCs because they can adapt to new situations quickly and are thus often claimed to be engines of innovation and growth (Bak et al. 2020; McGuiness et al. 2018; Organization for Economic Co-operation and Development (OECD) 2020a). Nevertheless, due to their limited access to financial and operational resources as well as to skills and management capabilities (Bak et al. 2020; Caniato et al. 2016; El Baz and Ruel 2021), SMEs always face the danger of becoming bottlenecks instead of drivers of economic progress (Bak et al. 2020; Kalemli-Ozcan et al. 2020; Klyver and Nielsen 2020; OECD 2020a/b; Thorgren and Williams 2020). This research gap has been amplified by the COVID-19 pandemic (Bak et al. 2020; El Baz and Ruel 2021; Ivanov and Dolgui 2021; Quayson et al. 2020; van Hoek 2020).

The solutions and the supporting factors of organizational and SC resilience that have been examined in the literature thus far include the use of new technologies, such as blockchain or artificial intelligence, procurement and shipping strategies, and the role of co-operation (for an overview, see the literature review of Baryannis et al. 2019; Belhadi et al. 2021; Dubey et al. 2020). Co-operation, which is also defined as SC collaboration, is seen as a crucial factor for SC resilience. Collaboration depends on the existence of mutual trust between the SC partners (Dubey et al. 2021; Duong and Chong 2020; Scholten and Schilder 2015). Some have claimed that the literature still fails to establish the precise manner in which collaboration makes companies and networks resilient (Folke 2006), especially when it comes to the topic of SMEs (El Baz and Ruel 2021; Ivanov and Dolgui 2021).

Since the possibility of regulating all contingencies through a contract is limited (Williamson 1979), formal mechanisms must always be supported by informal ones. For the purposes of this article, informal mechanisms are defined as relational. The means of combining them to make an SC resilient have been subjected to little academic scrutiny, especially in the context of SMEs (Duchek 2020; El Baz and Ruel 2021; Ivanov and Dolgui 2021; Wieland and Durach 2021). Companies within an SC usually have to manage two types of partnerships, an upstream relationship with suppliers and a downstream relationship with customers. For this reason, the combination of contractual and relational mechanisms that are employed in the two types of partnerships need to be analyzed.

This article uses a multiple case study approach (Eisenhardt 2021), in line with Duchek’s (2020) call for the wider use of observational methods in the study of the mechanisms that underlie organizational resilience. Chowdhury et al. (2021), in their literature review, reported that empirical research on SC resilience in the context of COVID-19 was scant, a finding that was further supported by Ivanov and Dolgui (2021). Bak et al. (2020) called for more empirical research on collaboration and contractual agreements. That research should consider the limited power that SMEs enjoy in SC relationships. Research on the means of overcoming asymmetric information and establishing trust between partners is said to be necessary to cope with crises (Duchek 2020; van Hoek 2020). Therefore, this article addresses two research questions (RQs):

### RQ1

How do SMEs combine contractual and relational investments to create resilience?

### RQ2

How does the combination of contractual and relational investments differ between customer and supplier partnerships?

Following Folke (2006) and Walker (2020), in the present paper, resilience is treated as a process rather than as an outcome. Accordingly, the results are analyzed on the basis of the conceptual model of organizational resilience that was introduced by Duchek (2020). The paper is premised on the assumption that trustful relationships must be established before a crisis (Blome and Schoenherr 2011; Duchek 2020; Pal et al. 2014; Ren et al. 2011). For this reason, this article refers to contractual and relational investments rather than to mechanisms. Since we conducted interviews in 2018, long before the COVID-19 crisis, and in April and December of 2020, during the first and the second lockdown, we were able to analyze the manner in which SMEs’ contractual and relational investments in SC partnerships laid the foundation for anticipating and managing the situation as well as for the adaptations that followed.

Our study is the first to offer empirical insights into the manner in which SMEs design and develop their customer and supplier partnerships and into the question of whether these measures enable SMEs to stay resilient. In addition, by virtue of adopting a longitudinal approach, we arrived at results that indicate which combinations and approaches have been most successful. In this way, we can offer advice to the managers of SMEs and larger companies on the means of establishing partnerships with such companies within their respective SCs. These findings concern real-world developments. We arrived at them in the middle of one of the gravest crises in history, and they are sure to benefit both research and practice.

The remainder of the article is organized as follows: Sect. 2 introduces Duchek’s (2020) model as the basic framework of the study. This introduction is followed by a definition of contractual and relational investments and an explanation of their connection to SC flows. After a brief overview of methodological matters, the sample, and the research process in Sect. 3, the RQs are analyzed and discussed by reference to the interviews in Sect. 4, which yields an adapted framework. Finally, the conclusion is presented in Sect. 5, which also offers an overview of limitations, avenues for future research, and managerial implications.

## Theoretical Background and Research Framework

Walker (2020) and Folke (2006) posited that resilience is an “offensive response to unexpected events” (Duchek 2020, p. 223) and thus accounts for the capability of a company to adapt to disruptions and crises by initiating change. Resilience is therefore a process rather than an outcome. This article therefore uses Duchek’s (2020) model of organizational resilience as the basis for the analysis. It not only reflects this definition, but due to the components it contains, it is also particularly suitable for the analysis of SMEs. The introduction of the model (Sect. 2.1) is followed by a brief introduction to the three SC flows and a definition of contractual and relational investments for the purposes of the study (Sect. 2.2). This sections also offers a detailed explanation of the constructs that we decided to use and the manner in which we combined them within the framework as basis for the case study.

### Organizational Resilience as a Process

**Fig. 1:**
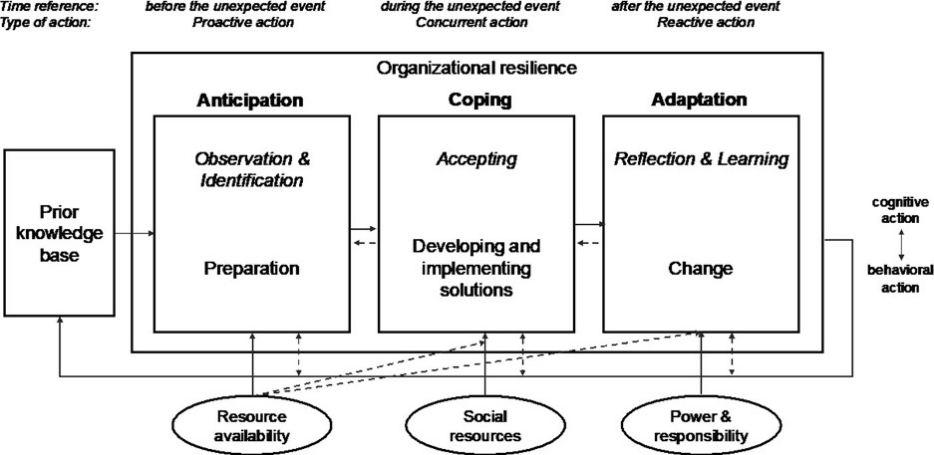
A Capability-Based Conceptualization of Organizational Resilience (Duchek 2020, p. 224)

Drawing on an analysis of the literature on process-based studies of organizational resilience, Duchek (2020, p. 224) proposed a model that is called “the capability-based conceptualization of organizational resilience.” As Fig. 1 demonstrates, it is a process with three main stages, namely *anticipation, coping,* and *adaptation*. The stages are influenced by *prior knowledge, resource availability, social resources*, and *power and responsibility*. The components are described on the pages that follow.

*Anticipation* is the ability to scan the environment continuously and to find means of accessing information. Anticipation has to do not only with market research but also with information sharing between contacts or within partnerships. According to Duchek (2020), companies that excel at anticipation also invest in human and technological resources, for example by training their employees for crises and by conducting planning exercises. SMEs are often incapable of making such investments due to financial and workforce limitations.

*Coping* is defined as accepting reality. Thus, the organization strives to find solutions while remaining sensitive to its limitations. The personality of decision-makers plays a crucial role, especially when resources are scarce. Coping also often entails improvisation. Duchek (2020) also wrote of ad hoc networks that are established to combine diverse sources of knowledge in order to find solutions, which must then be implemented rapidly. As mentioned previously, capabilities and partnerships that have not been established during the anticipation phase are often impossible to form at a later stage (Duchek 2020). This feature of the problem means that coping capability depends on past events, while the way in which a company organizes its coping phase affects its *adaptation capabilities* after the crisis. Companies should therefore take time to reflect and to learn from their processes in order to accumulate the knowledge that they need to meet future challenges.

Evidently, Duchek (2020) identified three influential drivers of resilience: *resource availability, social resources*, and *power and responsibility*. *Resource availability* refers to the time as well as to the financial and human resources that the organization invests in forecasting activities, but that also serve to create a buffer during the coping phase. For an SME, spare resources and parallel processes are seldom available because the balance between the costs of redundancy and the potential security benefits is difficult to strike.

Duchek (2020) also found another factor to be important. According to the model, it is only applicable to the coping phase. The *social resources* of an organization include the sharing of information, resources exchange, and cross-collaboration with other companies. The author conceded that mutual trust and respect need to be established during the anticipation phase, or even before it, for such social resources to be available. As far as *power and responsibility *are concerned, Duchek (2020) posited that they are needed only for adaptation, referring to decision-making processes that should not be based on hierarchy but on experience and expertise. The human factor thus remains relevant across drivers and stages.

Duchek (2020) further mentioned the continuous interplay between the cognitive and the behavioral actions of the decision-makers (see Fig. 1). Experience, expertise, as well as the relationships that decision-makers establish with other stakeholders within and beyond the industry should thus not be undervalued. The foregoing means that the model is relevant to the analysis of SMEs, in which the CEO or owner-manager is usually also the only decision-maker, or at least the main one (Arendt and Priem 2005). In sum, resilience capabilities are “deeply embedded in social contexts,” making them complex phenomena (Duchek 2020, p. 234). Due to its processual orientation and the inclusion of formal and informal mechanisms, the framework of Duchek (2020) provides a valid basis for the analysis of our interview results, which concern SMEs and COVID-19.

### Contractual and Relational Investments in SC Flows

Drawing on a review of the literature, Stock and Boyer (2009, p. 706) defined supply chain management (SCM) as the “management of a network of relationships within a firm and between interdependent organizations (…) that facilitate the forward and reverse flow of materials, services, finances and information from the original producer to final customer with the benefits of adding value, maximizing profitability through efficiencies, and achieving customer satisfaction.” Thus, three flows must be managed across an SC and by each partner individually, namely *product, financial,* and *information flows* (Stock and Boyer 2009).

Usually, products and financial resources flow in opposite directions, but they are strongly connected (Shi and Mena 2021; Wuttke et al. 2013). Contracts define who needs to produce and deliver what type of product, in what amount, to whom, and when. In addition, payment terms are agreed contractually between partners. Since suppliers often need to be paid early and customers often pay late, the resulting time gap, called the cash-to-cash cycle, needs to be covered financially so that materials can be produced and products shipped (Shi and Mena 2021; Wuttke et al. 2013). This financing operation is particularly challenging for SMEs, which usually do not have exceptionally strong financial foundations. Several contractual mechanisms that can solve this challenge have been developed and analyzed by practitioners and researchers over the last decades. Such efforts are usually said to pertain to the discipline of supply chain finance (SCF; Wuttke et al. 2016; Xu et al. 2018).

Contracts can only define what information needs to be exchanged to a limited extent. Information can be external (e.g., societal, political, legal, and industrial) or internal (Park et al. 2017). It can also be formal (e.g., facts and figures about logistic flows, inventory statuses, and production) or informal (Duchek 2020). The term ‘informal information’ refers to the experiences of the partners, which are often subconscious, and their dissemination depends on trust (Boone et al. 2019; Fu et al. 2017). Analyses of the means of defining this type of information flow are rare (Cruz and Liu 2011; Gunessee and Subramanian 2020; Gupta et al. 2020; Kumar et al. 2020). This said, timely information and predictions become assets, especially in pandemics (Boone et al. 2019; Fu et al. 2017). Differences in the status and quality of information mean that each partner has different opportunities to prepare for and survive a crisis (Zhao and Huchzermeier 2015). Several researchers (Bak et al. 2020; Gurbuz and Ozkan 2020) have found that SMEs are particularly unlikely to make such investments, mainly due to resource limitations.

Contracts can help to reduce complexity (Simon 1962) and are used to “organize transactions so as to economize on bounded rationality while simultaneously safeguarding them against the hazards of opportunism.” (Williamson 1998, p. 32) However, especially in the context of information flows, accounting for all future contingencies through a contract is not possible (Williamson 2007; Ren et al. 2011). As Zhao and Huchzermeier (2015) explained in their risk-management framework, it is the combination of contractual and relational factors within partnerships that is crucial to navigating a crisis. The veracity of this proposition has been demonstrated by several researchers (Cruz and Liu 2011; Duchek 2020; Grunert and Norden 2012; Rezaei et al. 2015; Wuttke et al. 2013; Zhao and Huchzermeier 2015).

Relational and contractual factors can be mutually reinforcing. For example, Liu and Cruz (2012) conducted a case study on South Korean companies and found that contractual foundations support closer collaboration and foster innovation as well as the development of trustful partnerships over time. Therefore, in uncertain environments, relational considerations are assumed to be at least as important as contractual ones (Cruz and Liu 2011; Pal et al. 2014; Poppo and Zenger 2002; Ren et al. 2011). In SCs, contractual and relational agreements have been found to be complements rather than substitutes (Poppo and Zenger 2002; Ren et al. 2011; Wuttke et al. 2013).

**Fig. 2:**
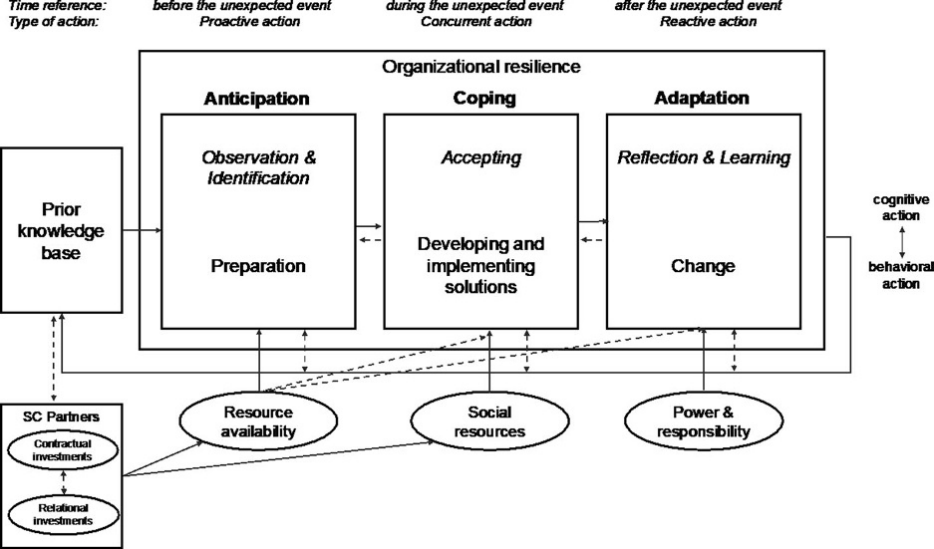
Research Model (adapted from Duchek 2020, p. 224)

In line with Ren et al. (2011), we define the contractual aspects of a relationship as formalized and often legally binding agreements. The term ‘relational aspects’, in contrast, refers to “embedding private and public information flows in a matrix of social ties.” (Ren et al. 2011, p. 731) In line with Duchek’s (2020) research model, we define contractual and relational investments in SC partners as antecedents and as influences on the drivers of resource availability and social resources (see Fig. 2). They can thus be seen as parts of the prior knowledge base. At the same time, resource availability and social resources can be enhanced by investments before and during a crisis. In addition, as Fig. 2 demonstrates, they influence each other. Table 1 (Appendix) overviews several examples of contractual and relational investments for each SC flow.

## Methodology: Case Study

Research on resilience of SMEs, especially in the context of COVID-19, is still at an exploratory stage (Chowdhury et al. 2021; McGuiness et al. 2018). An analysis of the 2006–2019 period reveals that 58% of the 101 articles from the sample of Bak et al. (2020) were case studies, surveys, or interviews. The other researchers who developed case studies on SMEs as parts of SCs before COVID-19 include Agostini et al. (2015), who wrote on strategic multi-partner SME networks, and Wuttke et al. (2013), who wrote on financial SCM. The contributions that demonstrate the usefulness of this methodology for analyzing the RQs of the present study are Benitez et al. (2020), with their longitudinal case study on the innovation ecosystems of SMEs in the Industry 4.0 era; Blome and Schoenherr (2011) with their study of SC risk management during the financial crisis; and Zhou and Li (2020), who found that information sharing has a positive effect on supplier investment and firm performance at SMEs in China. The topic is thus suitable for the application of a multiple case study approach, as indicated by Eisenhardt (1989, 2021). That method is said to yield generalizable findings and to open new lines of inquiry (Eisenhardt and Graebner 2007), particularly for RQs that focus on processes (Eisenhardt 2021). After an overview of the study design and a detailed description of the companies in our sample, the following section provides insights into the data collection and analysis on which the results were derived.

### Research Design, Case Selection, and Description

The inductive approach of Eisenhardt (1989, 2021) is expanded by a contextual one, as proposed by Welch et al. (2011, 2022). According to the authors, context is also useful as an explanatory factor. Duchek (2020), Wieland (2021), and Folke (2006) saw its importance in resilience research. Bak et al. (2020) cited Hooks et al. (2017, p. 2), who wrote that “resilience can only be truly tested in times of adversity or crisis.” In this way, the authors underlined the proposition that the context in which an SME operates has a significant influence on its resilience (Bak et al. 2020).

COVID-19 thus provides the context of this study. It serves an endogenous factor. As far as our general methodological approach is concerned, we followed Eisenhardt (1989, 2021). We arrived at our sample by using a theoretical sampling approach (Eisenhardt and Graebner 2007), that is, by screening official government databases and by contacting experts and business contacts. Following Eisenhardt (2021), we chose cases in which we expected to find contractual-relational combinations as bases for organizational resilience. We used the similarities and the differences between the cases to build a superior theory (Eisenhardt 2021). Another variable that we used was activity in business-to-business (B2B) contexts. The relationship-specific investments that are needed for business-to-consumer (B2C) operations differ from those that are needed in B2B contexts and require individual analysis (Lilien 2016).

We expected that restricting the sample to a single industry or a single type of product would reduce generalizability, but we also assumed that focusing on a single country would be important. Legal and financial circumstances, including lockdown strategies, vary across countries, making generalizability across national contexts a point of difficulty (Chowdhury et al. 2021). The absence of complex external influences is conducive to literal replication (Da Mota Pedrosa et al. 2012). Researchers tend to focus on China and the US when studying the impact of COVID-19, which leads to a gap in the literature. According to the literature review of Chowdhury et al. (2021), 99.8% of companies in Europe in 2020 were SMEs, which means that such organizations are the backbone of the European economy (European Commission, 2021). SMEs from Germany, a country with a strong SME tradition (Audretsch et al. 2018), are reliable business partners across the globe (Heider et al. 2021) and benefitted from exceptional government support during the COVID-19 pandemic (Kalemli-Ozcan et al. 2020; OECD 2020b, c). For this reason, the present study focuses on a sample of German SMEs.

We defined an SME as a company with fewer than 250 employees, in line with the OECD (2005) and the European Commission (2021). This said, some of the companies in the sample had larger global workforces, as noted in brackets in Table 2 (Appendix). This led to the turnover of the sample companies often being higher than the threshold of €50 billion (European Commission 2021; OECD 2005). The interviewees preferred not to indicate how turnover was apportioned between subsidiaries or jurisdictions. Poor alignment with the criteria of workforce and turnover is a specific issue for German SMEs, as noted by Heider et al. (2021). It is for this reason that the SME sector in Germany is often defined as *Mittelstand* by reference to qualitative criteria, such as family ownership, owner commitment, and continuity, meaning that even multinationals may be described as SMEs. Furthermore, the German mother companies met the OECD (2020b) criteria for SME status. For these reasons, they were included in the analysis.

The sample includes eight companies from seven industries (see Table 2, Appendix) with diverse finance policies, ownership structures, and roles within SCs, ranging from original equipment manufacturers (OEMs; Company B, Company C, Company E, and Company F) to crucial suppliers of parts (Company D) with combined services and logistics (Company A and Company G) as well as a third-party logistics (3PL) provider (Company H). Company E went bankrupt two years before our first interview round and was purchased by a private-equity company. Furthermore, when we conducted the second round of interviews in 2020, Company D had just filed for bankruptcy due to the COVID-19 crisis. Company E sought bankruptcy protection again in 2019, making a second interview impossible. Nevertheless, we decided to include both companies in our analysis because cross-case examinations can shed light on differences in precrisis relational and contractual investments. Furthermore, the variety of the sample enabled us to concentrate on abstract and general issues of contract and relationship design. We were able to determine which contractual and relational investments were used to manage the crisis independently of context-specific considerations such as the product, the position of the company in the SC, or its industry.

### Data Collection, Interview Approach, and Data Analysis

**Fig. 3:**
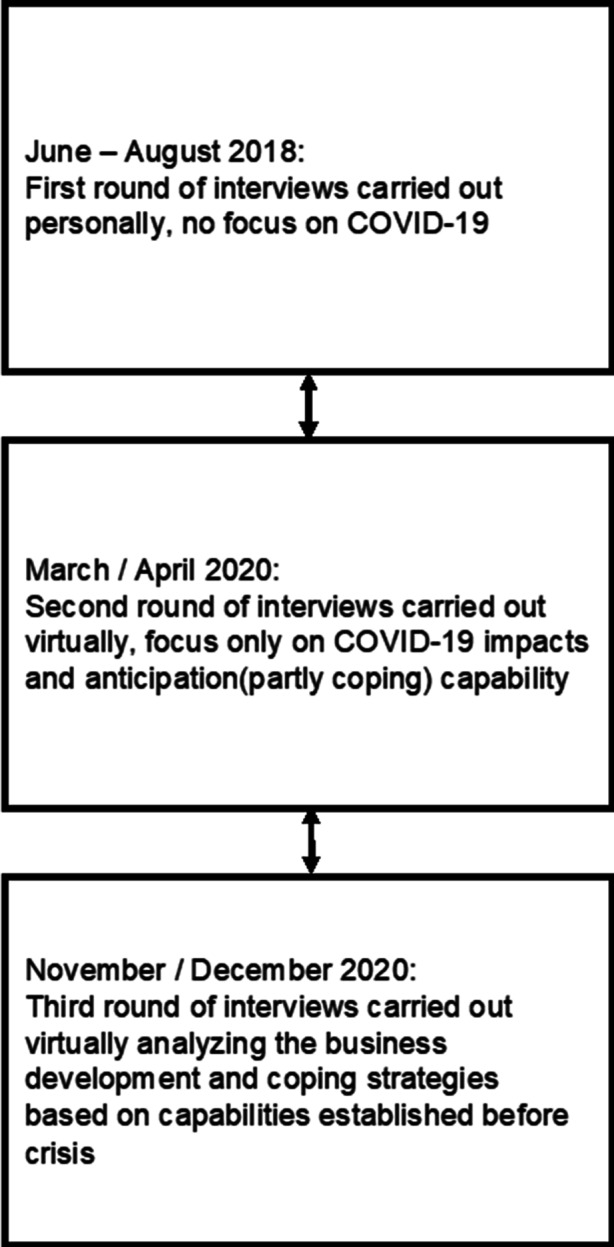
Overview of Interviews

We conducted interviews with key personnel from finance, control, purchasing, and SCM departments on three occasions over a period of two years. While the focus during the initial interviews, which we conducted between June and August 2018, was on product, financial, and information flows in general, it changed for the March and April round of interviews as well as for the last one, which took place in November and December 2020. In the later interviews, we focused on anticipation and coping on the basis of flow definitions that predated the crisis (see Fig. 3). The interviewees were assumed to possess the most strategic vantage point on SC flows due to their hierarchical and professional positions within each SME. At four companies (Company A, Company D, Company F, and Company G), it was only possible to speak to the CFO or the CEO (Company A). Two companies (Company D and Company F) had adopted a tight reporting structure in which financial and operational processes combined were combined under the exclusive control of the CFO. The third company (Company G) was the largest in the sample, but it also had the simplest operational structures. Company A was a fourth-generation family business, and its CEO participated in each process.

To maintain internal validity for these companies, we focused on secondary data from sources such as company documents, presentations, and reports, which we requested from all companies or acquired ourselves in order to minimize bias and ensure triangulation (Meredith 1998; Goffin et al. 2019; Eisenhardt 2021). Furthermore, we met the participants in person during the first round of interviews (we used video conferencing technology for the second and the third round of interviews). In this way, we gained a better understanding of their nonverbal behavior and could establish personal relationships with them. By combining the answers from each interview round with additional qualitative and quantitative data at each stage, we arrived at results that demonstrate which company could navigate the crisis best on the basis of its contractual and relational investments in its partners.

The interviews were based on a semi-structured questionnaire, which, in turn, is based on a case protocol that covers matters such as data collection, data access, and questions. This particular type of questionnaire is flexible, and questions can be adapted to the interviewee’s role and perspective as necessary (Da Mota Pedrosa et al. 2012; Eisenhardt 2021). Open and probing questions also yield more accurate answers (Eisenhardt and Graebner 2007). The 2018 interviews lasted between 90 and 120 min, while those that we conducted in 2020 lasted between 45 and 60 min. They were all transcribed and sent to the interviewees for review. Some interviewees were contacted again to answer additional questions that emerged during coding and transcription. The final report was then discussed with some of the participants, who were given opportunities to provide feedback and to validate the findings. We closed the sample as soon as additional interviews stopped yielding new information. External validity can be observed in the replication logic that is used across the cases, which were chosen because they were expected to support the theoretical foundations that we identified in the extant literature. We ensured reliability by using a case study database and interview protocols.

We adopted an iterative and inductive process of theory development (Eisenhardt 1989, 2021). We conducted a within-case analysis first. This was followed by a cross-case analysis, in which we compared cases by using different techniques. We examined the match between the data and the literature often, and we noted additional insights. Furthermore, following Welch et al. (2011, 2022), we considered the context of our analysis. We ensured internal validity through pattern matching and evaluation building. To that end, each of us created a coding tree. Subsequently, we conducted in-depth discussions and comparisons in order to distill a definition that would explain the phenomenon under observation best. We mapped the data into categories, which allowed us to position the companies within our framework. An overview of reliability and validity orientations is presented in Table 3 (Appendix). The deep conceptual interpretation of the phenomenon yielded the final framework, which is presented in Sect. 4.4.

## Results, Analysis, and Discussion

For both partnerships, the way the sample companies combined contractual and relational investments is analyzed in the following, each resulting in a graphical representation. Based on this, we show in Sect. 4.4 to what extent this has affected the anticipation and coping ability of each company. This chapter ends with the answer to our RQs resulting in an adapted framework that links Duchek’s (2020) process to our results.

### Contractual and Relational Aspects of Customer Relationships

Most companies focused on “combining individualization for customers while still keeping economic feasibility due to high lot sizes or regular transactions,” as the CFO of Company F put it. On the one hand, companies combine contractual and relational investments in customer relationships to ensure resource availability by guaranteeing regular incoming payments from product sales. On the other hand, customers, as sources of information or investment in the co-creation of products, enable companies to gain social resources (Duchek 2020). While Company A, Company E, and Company F focused on providing a standardized product that could be tailored to customers, Company C, Company G, and Company H only sold tailored products and therefore concentrated on specific contracts with each of their customers. This strategy, of course, calls for investments of time and human resources as well as for production capacity. The CEOs of these companies saw such investments as bases for social resources for the reasons mentioned above.

Company B and D additionally offered possibilities of joint development. Company B, acting as a wholesaler and thus not as a producer, concentrated on individualized bundles of products that it adapted to the expectations of customers. Furthermore, it provided training of employees at customer sites as well as the provision of joint development opportunities, which required significant investments. Company D also offered joint development, but the content of the contracts was dictated by customers, and there was thus no proactive investment in relationships. In some cases, Company D did not enter into contracts. Instead, it would provide more labor than it had agreed contractually, which, unfortunately, was not valued by its customers.

The data demonstrates that the forms of financial investment into a partnership range from the use of pure financing instruments, such as trade credit, to the application of more relationship-specific techniques, such as individualized payment terms or discounts that depend on the value of a customer. In general, Company D, Company G, and Company H only adopted payment terms that they negotiated individually with each customer, while half of the companies in the sample (Company A, Company B, Company C, and Company F) only offered such terms to highly trusted and regular customers. Those companies that differentiated between customers used other instruments to ensure that financial resources were available, such as high equity or SCF. Company C and Company F, for example, used SCF actively, whereas Company B used SCF passively and only as a safeguard. However, Company B stated that it had increased its use of SCF during the crisis. Thus, relational investments in customer relationships were often safeguarded by contracts and supported further by other financial tools that reflected the financial base of the SME.

The two companies that had the lowest amount of financial resources used techniques that were completely different from those that were employed by the others. Company D was sacrificing profits by accepting unfavorable payment terms or by offering technologically intensive product individualization. It further stated that it would often sell its products at prices that were too low to ensure its survival, which coheres with the findings of Liu and Cruz (2012) and McGuiness et al. (2018). Company E adopted a wide range of measures to satisfy customers financially, but it failed to offer a convincing product. Its suppliers developed their business models over time and were ultimately capable of providing finished products themselves, which led to the bankruptcy of Company E. According to the interviewees, Company D and Company E engaged in fewer relational investments because they lacked contacts. Customers had no interest in investing time in Company D and Company E, which demonstrates that a financial basis, which may comprise equity or bank loans, is crucial to relational investments in customer partnerships.

Those who could establish extracontractual relationships with their customers did so primarily through information flows. A mixed approach predominated in those cases as well. Company F was an exception. The interviewee from that company mentioned that, due to its market positioning, the company would only invest in some of its largest customers, but that “this is an area to focus on in the future.” In contrast, Company A, Company G, and Company H engaged in information flows and tailored them intensively, meaning that they adopted more relational techniques than contractual ones. The CEO of Company A would visit customers regularly, support them during events, and provide assistance with sales, leading to a relationship where “we see them more like friends than business partners.” Company G did not usually enter into contracts because, according to its CEO, “if the relationship does not work, the contract would not help anyway. Investing in the relationship is way better than investing in legal consulting and contracts.” Furthermore, the CEO in question was highly active in industry and politics, which enhanced the knowledge base of the company, thus preparing it for anticipation and coping, in line with Duchek’s (2020) theory.

According to the CEO of Company H, “people are most important,” and the company would “always communicate openly and make failures transparent.” The implication is that the company focused strongly on personal relationships. Furthermore, “our customer is the customer of our customer, so we need to focus on them to offer benefits.” This practice meant that customers would depend on Company H to a larger extent. The CEO and his direct reports would engage in personal contacts with customers and in regular exchanges about markets, politics, and fluctuations in demand. They would also offer individualized product tracking solutions. This service could only be provided due to the availability of resources such as money, time, and IT capabilities.

Company B and Company C also relied on information flows in customer relationships. They offered training and consulting, which helped them to understand customer needs better. According to one of the respondents from Company B, “It also makes our customers dependent, if not knowledge-wise, then emotionally.” In addition, the company would attempt to offer information that fell outside of the scope of contracts to its customers in order to provide them with knowledge that they would not otherwise obtain. This offering implies that the company could obtain information of this kind from appropriate sources, which, as the interviewees stated, were often suppliers. Not all SMEs have such capabilities and or access to such sources.

**Fig. 4:**
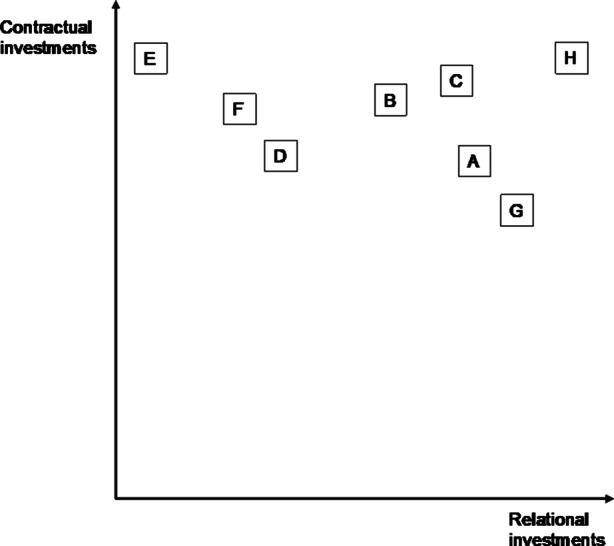
Combination of Contractual and Relational Investments in Customer Relationships

In summary, as Fig. 4 demonstrates, the results revealed highly diverse customer relationships. The actual contractual and relational investment activities for each flow are described in detail in Table 4 (Appendix). Relational investments were defined as crucial by all interviewees. Only sufficiently capitalized SMEs could engage in them. The companies that made relational investments in customers extensively could always rely on a strong basis of contracts or financial safeguarding tools.

### Contractual and Relational Aspects of Supplier Relationships

All SMEs had strong contractual partnerships with their suppliers. The respondents considered having more than one supplier of basic or rare materials to be important. Such arrangements would decrease dependency on suppliers, which are often more powerful than SMEs (Bak et al. 2020; Ren et al. 2011), which according to Duchek (2020) is also crucial for resource availability. As noted previously, the suppliers of Company E became its competitors. Payment and information flows had to be organized rather than standardized and were contractually defined by suppliers. Company D also had the advantage of being able to offer crucial knowledge and to establish joint R&D programs as well as cross-investments with suppliers, but its knowledge, finance, and human-resources endowments proved insufficient for survival, unlike those of other companies.

**Fig. 5:**
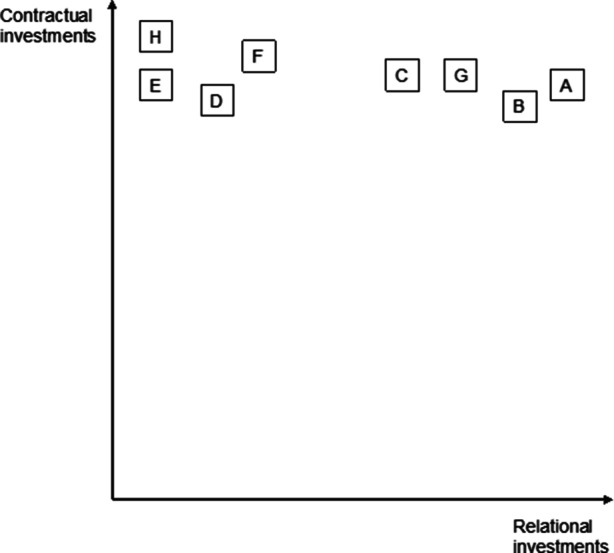
Combination of Contractual and Relational Investments in Supplier Relationships

For Company F and H, the supplier dependence was rather low, leading to a different partnership design. Company F produced most of the materials that it needed in-house and thus only needed certain basic materials, for which it negotiated individual contracts and payment terms. Company F had such a low dependency on suppliers that the CFO would only engage in exchanges with them on the occurrence of market and industry developments. As in the case of customer relationships, the interviewee from that company mentioned a plan to focus on these partnerships in the future. Company H, which only needed basic products that could be sourced from various suppliers, would only invest in quality assurance. Such agreements would be agreed contractually and in advance. The variability of supplier-relationship designs was thus relatively low, compared to that observed for customer relationships, and contractual investment predominated (see Fig. 5).

Company G also engaged in relational investments. The CEO of Company G, which, similarly to Company F and Company H, did not depend on its suppliers, used quality assurance meetings to exchange information about the industry and politics with suppliers and used them as sources of information to enhance the social resources of the organization. This approach may be contrasted to that of Company H. Furthermore, the personal visits were a form of investment in the relationship, and Company G was seen as a reliable and trustworthy partner.

This was also the case for Company A, Company B, and Company C, which invested extensively in personal relationships with suppliers. Company A, although it did not need any rare ingredients and had adopted a multi-supplier strategy, concentrated on relational investments that were based on the standardized contractual agreements into which it entered with suppliers. The CEO and a large purchasing team would visit suppliers regularly to exchange information about quality, the global industry, and the political circumstances of each country. Company C used rare and important materials whose prices are not negotiable because they depend on the markets. The CEO and the Head of Purchasing would exchange information with suppliers in order to understand and anticipate market fluctuations. They also offered joint R&D initiatives to individualize products and to enable suppliers to draw on their expertise. The focus was on informal information-exchange visits. The same practice had also been adopted by the C‑level team at Company B, whose raw materials came mainly from China. Contracts were negotiated over long periods of time and were designed to be in force for many years. Company B therefore also provided coaching and training as well as engaging in joint R&D with its suppliers. In addition, the CEO would visit suppliers personally and regularly, and they would invest time into socializing. The four companies thus invested extensively in relationships. The investments in question were both relational and contractual, irrespective of the scarcity of the materials that the suppliers provided and the corresponding allocation of power.

If they are to decrease opportunism and lead to beneficial transactions, investments must establish governance structures. This said, the partner first needs to be convinced that the transaction will benefit them (Williamson 1979, 2007). In some cases, suppliers only accept business at higher prices or with shorter payment times. In addition, prices are uncertain due to stock-market fluctuations and instability in the countries from which materials are sourced (Company A and Company C). The latter has increased during the pandemic. All companies except Company D and Company E would thus strive to be financially reliable partners. As the CEO of Company G said, “Suppliers can be sure that they get their money at the right time.”

Company B mentioned that this behavior was a crucial pre-investment that ensured that supplies would continue during the crisis. According to the CFO of Company B, “although we are not an important customer, we are a reliable customer and always hold our promises. This helps us to be valued by our suppliers and getting access to the material we need on time and in good quality.” Since instability increased during the pandemic, these investments were even intensified in an attempt to ensure supply. An interviewee, who was in charge of SCM at Company B, said, “Punctual payment helps us to differentiate from others, especially in Asia.” In the case of Company D, cross-investments with suppliers facilitated production, innovation, and quality but could not prevent bankruptcy. A similar approach might also have been open to Company E—its suppliers were also its strongest competitors. In 2018, the interviewee from that company mentioned that the failure to adopt the approach in question had been a strategic decision. The projection of financial reliability is thus supported not only by adherence to contractual agreements but also by the introduction of relational investments into the financial flow. Evidently, these techniques can only be employed by companies that rely on their own equity and monetary bases.

For most companies in the sample, suppliers were the best source of information about developments in the law and the markets and thus about forecasts, in line with the findings from literature (Heider et al. 2021; Zhou and Li 2020). They had therefore engaged in joint R&D to sell patents or products with their suppliers. By working so closely together, they had “made the partner dependent through the knowledge offered,” as the CEO of Company G said. Auditing, training, and consulting offers are another option. Company A would even organize events at its headquarters, including competitions between suppliers. Furthermore, it had invested in creating a spirit of togetherness across the whole upstream SC by introducing branding activities within a production team. As a whole, the SMEs in our sample provided more information and knowledge to their suppliers than what was required of them contractually, implying investment in social resources. The option of making such investments, however, was only available to companies that had sufficient financial capital, time, and human resources (purchasing teams) as well as competencies that suppliers could benefit from.

In addition, it should be mentioned that much depends on the type of supplier, the type of product that it offers, the country in which it operates, the resulting shipping costs, and payment process terms. There is a difference between companies that depend on their suppliers and companies that do not. The latter base their relationships on contracts while making limited relational investments to obtain important information and knowledge. In contrast, relational investments are crucial for companies that depend on their suppliers. The most striking example is that of Company A, which treated its interactions with numerous suppliers as an opportunity to create an asset, that is, a large team characterized by mutual trust, on which it could rely during the pandemic. Detailed examples of contractual and relational investment activities for each flow are provided in Table 4 (Appendix). Fig. 5 displays the predominantly contractual technique of designing partnerships with suppliers, with Company A, Company B, Company C, and Company G also engaging in relational investments.

### Impact of Contractual and Relational Aspects on Anticipation and Coping

This subsection focuses on answering the two RQs, which inquire whether the various combinations of contractual and relational investments into SC flows generated sufficient (social) resources to anticipate the COVID-19 pandemic and to cope with it. In addition, the findings from the sections before are synthesized in order to summarize the observed differences between customer and supplier partnerships. Adaptation is not analyzed further because the interviews were conducted during the pandemic. However, some outlooks are identified.

#### Anticipation

During the second round of interviews, which took place at the start of the pandemic, the interviewees from all of the organizations except those from Company D and Company E stated that they felt ready for what was to come. Some even said that they had known about it well in advance. At that time, Company E had filed for bankruptcy and was in the middle of the liquidation process. Company D was already in distress, and its representative also indicated that they were surprised by the situation. As for forecasting and planning, Company A, Company B, Company C, and Company G stated that they had already prepared for a crisis at the beginning of the year, when COVID had only been detected in China, because they had received information from their suppliers. The interviewees from Company F and Company H mentioned that they had not anticipated the crisis but nevertheless felt prepared due to their ex ante relational investments. Preparation was thus evaluated as satisfactory, even though identification and anticipation might not have been complete.

The interviewees from Company A, Company F, and Company G felt prepared because they had accumulated large inventories, which they could use to maintain production. However, Company F, among others, saw its customers become less inclined to spend money on their products because furniture is something of a luxury product. Company A and Company G, being active in the food industry, had already begun to strengthen their contacts with suppliers earlier in the year in order to ensure that shipments would be as reliable as possible. They furthermore prepared several options for customers who were interested in extending payment times or adjusting orders. Since both of them had already invested extensively in trustful partnerships, not only in the SC but also with industry and market specialists, joint planning increased their social-resource endowment. These investments also showed how they had benefitted from their inventories, financial resources, and human capital. Human capital proved particularly valuable because sales and purchasing teams could be used to acquire the latest information from the countries in which the companies were active, enabling Company A and Company G to adjust their strategies and to identify scenarios for different parts of the globe.

The same was true of Company C, which transacted with public authorities and large corporations in the sports and leisure industry. The offerings of that industry was seen as a luxury during the pandemic. Due to its dependence on the commodity markets for raw materials, the company discovered that the hedging strategy that it had introduced previously was highly valuable. At the beginning of 2020, Company C had begun offering the individualized handling of existing contracts to customers in China, an approach that it later adopted across the globe. Once more, the sales team turned out to be one of the most important assets of the company. In addition, contracting with public authorities, who are generally reluctant to cancel contracts, improved the outlook of the company. Therefore, as far as resource availability is concerned, Company C could rely on a sound financial basis and on appropriate customer contacts and contracts. Furthermore, access to public officials enabled it to access more information.

Company B had the best anticipation opportunities and made use of them. Due to its heavy dependence on Chinese suppliers, it had sensed problems in shipping and material supplies as early as November 2019. Due to cultural and language issues, identifying the root cause of the problem took some time, but in January, its employees knew that a dramatic disruption was likely and had begun to plan for it to the best of their abilities. For that reason, they only faced a delay of four weeks at the beginning of the German lockdown. Thereafter, they could deliver their products to customers as agreed. The SCM team worked with suppliers to identify rail and road solutions because maritime transportation was problematic. In evaluating the anticipation capability of Company H, its CEO stated that he did not predict the developments early enough.

In summary, Company A, Company B, Company C, and Company G performed best, as far as anticipation is concerned, mainly due to their strong partnerships with suppliers and customers. These partnerships enabled them to acquire information early and to prepare strategies with their partners. The strong equity base that all of these companies could rely on was critical. It provided them with opportunities to invest in inventories and partnerships ex ante. Thus, anticipation requires partners and investments, which should be both relational and contractual. This said, contractual ones are never sufficient in isolation and always need to be accompanied by relational investments that are as extensive as possible.

#### Coping

As Duchek (2020) wrote, accepting a situation and finding solutions that are based on internal expertise and experience and which function across teams is an important step in the coping phase. In addition, it is always important to know the limits of the company in order to avoid embarking on changes that cannot be completed due to restrictions that are related to finance, capabilities, or other limitations. With the exception of Company D and Company E, the organizations in our sample made changes relatively rapidly, even though all of the interviewees stated that they could have never conceived of the situation that developed.

All of the respondents who had invested heavily in establishing and maintaining positive relationships with suppliers and customers before the crisis, mainly by demonstrating reliability, assumed that their actions would be conducive to resilience. The CEO of Company G said, “No poker, we are always a reliable partner.” Company B and Company C even mentioned that, before the crisis, they could often negotiate the terms that they needed. This did not change during the pandemic, and negotiations of this kind became more frequent. However, in such periods, the participants in SCs focus more on revenue than on risk mitigation. Therefore, information sharing is crucial, while improvements in material and financial flows become less important (Ma et al. 2020). For example, Company A, Company B, Company C, and Company G had increased their investments in the co-development of products with customers and suppliers in order to make rapid adjustments in crisis situations. For the two companies that were active in the food industry, this meant adjusting recipes on the basis of the ingredients that were available in their inventories or at the locations of their customers.

Furthermore, the existence of multiple supplier relationships that had benefitted from extensive investment transpired to be crucial, as noted by the interviewees from Company A and Company B. Company B also provided even more training to the employees of customers, as well as co-development and joint R&D efforts. Given the supply situation in China, it had to adjust its offerings on a relatively regular basis. The individual sales and purchasing teams of Company A, Company B, Company C, and Company G also demonstrated their value by offering customized support. Furthermore, team spirit, both within the SME and across the SC, improved, as reported by the interviewees from Company A, Company B, and Company C.

Anticipation facilitates coping, but coping can also occur without anticipation. For example, Company F and Company H turned out to be highly proficient at coping, which compensated for their deficient anticipation capabilities. Company H, as a 3PL business, offered individualized support to its customers proactively, which turned out to be its most important asset during the coping phase. Demand for online delivery was particularly high. The challenge that Company H faced was a shortage of human resources. The CEO therefore partnered with some companies from the industry network in which he was active in order to motivate employees to join his company on a short-term basis. Due to the involvement of the CEO in politics and industry, this recruitment drive succeeded easily and quickly.

At the beginning of the crisis, Company F struggled to cope, especially since it had not anticipated the pandemic and customers began to delay payments. The CFO, when we spoke to him in the course of the third round of interviews, had ceded to adjust the business model and to concentrate on the home-office trend, which increased demand. This move can also be seen as a first step towards adaptation. The company did not have to rely on too large a number of suppliers because most of its ingredients were produced in-house. Therefore, the shift in focus was easy to operationalize. The foregoing demonstrates that the availability of resources, especially financial ones, the competencies of the employees, and social resources are important for coping. We adjusted our research model accordingly, a point to which the exposition will now turn.

### Discussion of Results and Adaptations of the Research Model

Our results show that there are differences in the manner in which contractual and relational investments are combined in customer and supplier partnerships. For customers, most companies are located in the upper-right corner of the model, that is, they invest in both contractual and relational mechanisms (see Fig. 4). This said, contractual mechanisms become less important as the partnership develops and are often used only as safeguards. The variability of the relational organization of supplier partnerships is higher (see Fig. 5). Some companies only focus on contracts. Others see them as crucial but also engage relationally. In summary, half of the companies in our sample (Company A, Company B, Company C, and Company G) focused on relational investments in suppliers while also engaging in relational investments in consumers to a reasonable extent. Company F was rather reluctant to make either type of investment, while Company H invested extensively in customers and to a small degree in suppliers. Company D and Company E are special cases in our sample. They demonstrate that near-exclusive reliance on contractual partnerships upstream and downstream does not lead to resilience and that relational investments are only possible when the company offers a convincing product or asset to its partners.

**Fig. 6:**
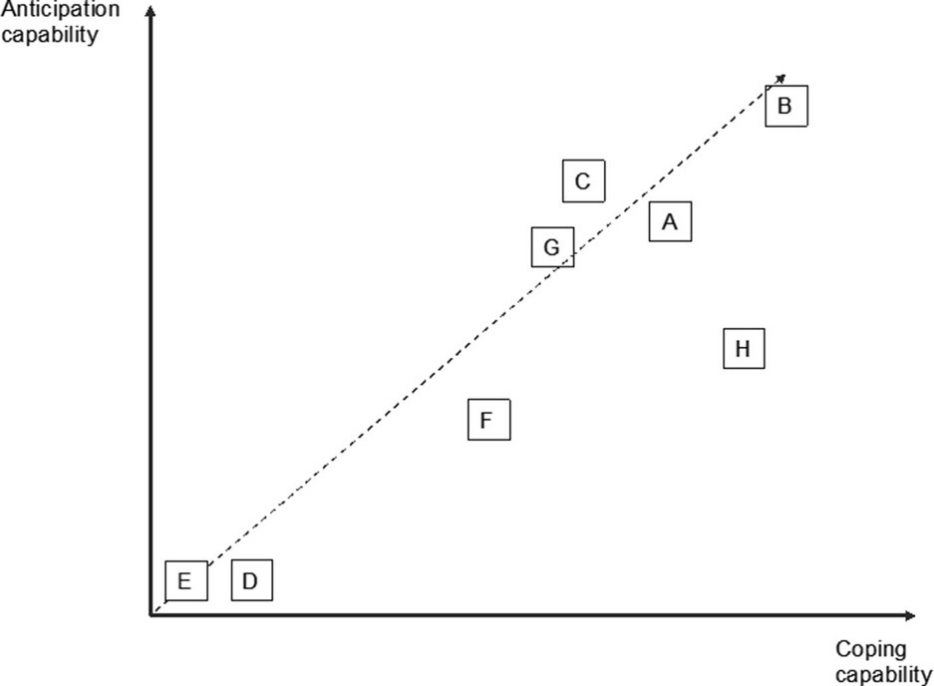
Anticipation and Coping Capabilities of the Sample Companies Leading to Resilience

Fig. 6 shows what these combinations mean for resilience. It is assumed that resilience is based on the ability to anticipate and cope—adaptation would not be possible otherwise. The dotted arrow shows the best-case scenario. As can be seen, Company A, Company B, Company C, and Company G are closest. While Company H and Company F did not foresee the crisis as early and as clearly as the others, they managed to cope with it successfully and had even begun adapting their business models and processes to the new circumstances, in line with Duchek’s (2020) model. Company F was exploiting the home-office trend, while Company H had expanded its workforce to meet large-scale online demand. However, given its lackluster anticipation capabilities, Company F might find itself becoming less resilient after another crisis, in particular if it fails to cope with new circumstances quickly.

It follows that the optimal means of achieving resilience is to engage in contractual and relational investments in both types of partnership. These results enabled us to answer the two RQs. RQ1 inquires how SMEs combine contractual and relational investments to anticipate and cope with crises. RQ2 focuses on the difference between customer and supplier partnerships in the context of this combination. We put forward two propositions on this basis.

#### Proposition 1

Resilient SMEs combine contractual and relational investments by using

- contractually agreed product flows, with a focus on quality and individualization in the execution of contracts;
- individually agreed contractual financial flows with additional relational investments; and
- relationally oriented information flows.

#### Proposition 2

The SMEs that navigate a crisis optimally focus on strengthening both types of partnership relationally while also safeguarding partnerships with suppliers contractually.

The propositions are supported by the literature. For example, several of the works that Chowdhury et al. (2021) reviewed indicate that relationships are crucial to safeguarding companies from the effects of crises (Hobbs 2020; Sharma et al. 2020). This said, three studies in their sample report that COVID-19 led to less interaction and thus to reduced information flows (Baveja et al. 2020; Gunessee and Subramanian 2020; Kumar et al. 2020). Pal et al. (2014), in their analysis of SC resilience, found that relational networking supports flexibility and rapid decision-making as well as the effective management of material and financial flows. Gupta et al. (2020), however, wrote that increases in opportunistic behavior and reductions in collaboration, as well as diminishing supplier engagement (van Hoek 2020), mean that even the most positive relationships ought to be combined with strong contracts (Gupta et al. 2020).

Most companies thus safeguard financial and product flows via contracts and focus on relational considerations in the information flow. Since SMEs often cannot invest into information-generation activities and technologies as heavily as larger companies, partnerships provide a crucial foundation for their resilience (Grunert and Norden 2012; Heider et al. 2021). Suppliers are the more important building blocks of that foundation, as shown by our results. The foregoing also means that investment in human resources such as sales or purchasing teams, which negotiate and personalize contracts and form the relationships in question, is of considerable importance. As shown in Table 4 (Appendix), in some cases, the exact difference between a contractual and a relational investment in each flow depends on the general circumstances of the partnership.

The discrepancy between approaches cannot be explained by differences between industries or business models alone. The decision on how to design relationships also seems to be driven by the business philosophy of the owner and by the financial base of the company, which may comprise equity or contacts with banks. The nature of an SME may be an asset, as far as resilience is concerned—the lower complexity of SME structures and the concentration of decision-making power in a few individuals or a single person facilitates the rapid implementation of solutions. In particular, Company B, Company F, and Company H provided examples of products, contracts, and even business models being adjusted in accordance with the external situation and in close co-operation with customers and suppliers, which is another advantage of SMEs. In addition, it should be mentioned that, as B2B companies, the organizations that we studied benefitted from the fact that buying regularly from the same supplier reduces transaction costs for customers. SMEs that offer high-quality and specialized products and which can invest in partnerships with customers are especially likely to have little need for contracts as safeguards.

**Fig. 7:**
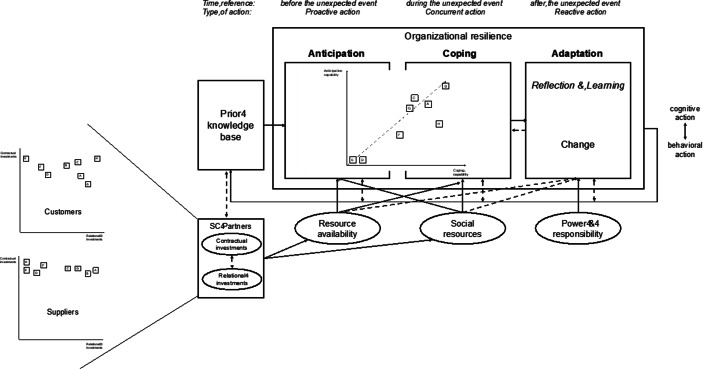
Final Framework Based on Study Results

Combining the results of our analysis and the figures presented above with the framework of Duchek (2020), we surmised that social resources influence the anticipation stage as strongly as they influence resource availability. Resource availability, in turn, influences coping. It is for this reason that we added arrows to Fig. 7, which may be contrasted with Fig. 1. Both are assumed to exert relatively indirect influences on adaptation, as can be seen from the dotted arrows in Fig. 7.

## Managerial and Theoretical Implications and Avenues for Future Research

As far as establishing business relationships with SMEs is concerned, our study provides some important insights for the managers of large corporations and SMEs. Financially constrained companies might find it desirable to opt for relational investments in order to increase mutual trust (Agostini et al. 2015). The empirical research showed that investments in training, coaching, individual customer support, products, and regular personal visits by the CEO and fixed contact persons are assets. Investing in relationships improves resilience. Duchek (2020) also characterized investments of time and human resource as crucial. SMEs at which the owner-manager is in a strong position rely on such investments to a larger extent than on contracts. The implication for larger companies is that creating business by being a reliable partner stabilizes SMEs and supports joint performance as well as SC resilience. Nevertheless, SMEs should ensure a secure financial flow, which can also entail relying on a sufficient equity base and on appropriate relationships with banks. Such practices also support the ability of the enterprise to invest in both types of partnerships by providing extracontractual benefits. This said, for suppliers, this decision should always reflect the balance of power between the partners.

Duchek (2020) wrote that it is human resources, such as sales and purchasing teams or CEOs, and their adroit application before and during a crisis that establish mutual trust in partnerships. The resilience of SMEs depends on the involvement of individuals. Trust and commonality of experience are necessary to cope and navigate a crisis together, and they can only develop between people.

Real-time information and relational coordination are vital, particularly in times of crisis when quick action is essential and forecasting is difficult (Duchek 2020). SMEs need support because they often possess limited resources and thus struggle to invest in new tools or to accept new partners. Improved access to information would offer SMEs a way of anticipating and coping with crises more adequately, both individually and as links of SCs. However, SMEs are currently reluctant to invest into new technological possibilities and instead rely on existing partnerships. In addition, the tools and technologies that they use need to be designed in a manner that enables smaller companies to purchase and use them efficiently. At the same time, these companies need to invest in parallel improvements to their processes and cultures (Gurbuz and Ozkan 2020).

We attempted to develop a profound analysis, and we conducted extensive research. However, this study is not free of limitations. Case studies, by their nature, only focus on limited amounts of data. Since our sample was highly diverse, in terms of the industries, strategies, and respondents that were included in it, and given that we drew on assorted secondary data, we are confident that our results can provide a basis for understanding the phenomenon under observation better. In addition, the influences that we found are quantifiable and can be tested on larger samples, which would make our study a springboard for future research. One interesting aspect, for example, would be more research on the perspectives of focal companies in these partnerships. According to Farahani et al. (2020), each disaster is different, which means that findings from one crisis might not be valuable in the next one. For this reason, more research on SMEs in other types of crises and from other industries and countries is required to obtain a more holistic perspective on overall SC resilience and the factors that contribute to it.

Our case study approach enabled us to understand the antecedents and the prerequisites for anticipating and coping with a global crisis among SMEs who participate in partnerships with suppliers and customers. We drew on the example of the COVID-19 pandemic. We interviewed participants from eight companies from different industries and found that the way in which SMEs design their partnerships before a crisis and their investments in those partnerships during its course have a significant impact on resilience. We contributed to theory and practice by answering our RQs with the propositions that we introduced in Sect. 4.4.

We found that most SMEs rely on contracts with their partners but also adopt numerous relationship-building measures, such as information flows. Contractually agreed partnerships are used more extensively in supplier relationships because, unlike in customer relationships, the power of SMEs is often limited. Since suppliers are often among the richest sources of information, SMEs improve their positions when they provide additional, individual, and oftentimes extracontractual benefits to them. Our study further demonstrated that the integration of real-time and behavioral data can support SMEs in improving their resilience. Williamson (1979, p. 260) wrote that technical change might “give way to more complex governance relations.” The same might be true of environmental or societal change, which can define new combinations of contractual and relational mechanisms that make SMEs, and thus SCs, more resilient.

## Appendix

**Table 1:** Examples of Contractual and Relational Investments

Topic	Examples (based on definitions from study)
Contractual Investments	*Product Flow*:
	Fixed or customized delivery times
	Fixed or customized lot sizes
	Defined quality of standardized products
	Defined individualization of products
	Defined context and tasks of partial joint R&D
	Timely delivery
	*Financial Flow*:
	Fixed prices per product or product lot sizes
	Agreed investments in special machinery
	Joint marketing and promotion
	*Information Flow*:
	Tracking of product/component location across SC
	Sharing of production status
	Collaborative planning, forecasting and replenishment (CPFR)
	Sharing of predefined industry and market data
Relational Investments	*Product Flow:*
	Joint innovation and product development/R&D
	Individualized products (beyond contractually agreed details)
	*Financial Flow*:
	Prepayments to suppliers (can be supported by SCF tools, especially if not agreed officially ex ante)
	Offering trade credits to customers
	Offering superior payment terms and extended payment times to customers (supported via SCF tools)
	*Information Flow*:
	Offering education/training/consulting to partners
	Regular audits of suppliers
	Regular personal and onsite meetings, especially visits to the partner’s location
	Inviting partners to events in order to support exchange
	Sharing informal information or expert knowledge
	Extracontractual sharing of political, industry, expert, and market data

**Table 2:** Sample Overview

**Company**	**A**	**B**	**C**	**D**
Industry	Food and nutrition/pharmaceuticals	Solar	Sport and leisure	Battery cells
Respondents’ Position	CEO	CEO, CFO, Head of Purchasing and SCM	CFO of holding, CFO of largest subsidiary, Chief Purchasing Officer	CFO
Turnover	€50–100 million	€240 million incl. all subsidiaries worldwide	€250 million incl. all subsidiaries worldwide	€80 million *bankrupt due to crisis in 2020*
Employees	220	150 (300 worldwide)	200 (550 worldwide)	50 (300 worldwide)
Ownership	100% family owned	100% family-owned holding with subsidiaries	Private-equity holding with subsidiaries and different brands	40% family owned cross-investment partners: 35% Chinese 25% South African
SC Role	*Crucial supplier* (production, logistics, sales, purchasing)	*OEM and wholesaler with one-stop-shop offering* (assembly only)	*OEM with one-stop-shop offering* (all parts of SC)	*Crucial supplier* (production only)
**Company**	**E**	**F**	**G**	**H**
Industry	Television technology	Business furniture	Food and nutrition	Logistics provider for several industries
Respondents’ Position	CFO	CFO	CFO	CEO, CFO, CEO of logistic subsidiary
Turnover	€138 million *bankruptcy in 2019*	€138.4 million incl. all subsidiaries worldwide	€565 million incl. all subsidiaries worldwide	€1 million/€4 million
Employees	200 (500 worldwide)	150 (550 worldwide)	80 (500 worldwide)	120
Ownership	Private equity	100% family owned, not managed by owner, holding company with subsidiaries and different brands	100% family owned, not managed by owner, holding company with subsidiaries	100% family-owned holding with subsidiaries large equity base
SC Role	*OEM* (assembly only, small R&D)	*OEM* *with one-stop-shop offering* (all parts of SC)	*Crucial supplier* *with one-stop-shop offering* (assembly only)	*Third-party logistics provider* *with one-stop-shop offering* **(**all solutions provided by the company)

**Table 3:** Measures for Ensuring Validity and Reliability (based on Eisenhardt 1989; Goffin et al. 2019)

Reliability/Validity Criterion	Research Phase			
	Design	Case Selection	Data Gathering	Data Analysis
**Reliability** (whether a study is replicable in that other researchers following the same steps would arrive at the same insights)	Derivation of a case study protocol and database	Selection of eight different cases of seven different industries based on careful defined and documented selection criteria	Shared and same questionnaire for all interviewers Shared case study database	Support from researchers not being involved in Data Gathering Independent coding by the research team individuals
**Internal validity** (whether the findings on causal relationships between variables identified through triangulation and pattern-matching are sound)	Profound literature review Use of a theoretical framework	Case study protocol with selection criteria	Selection of variety of interview partners and information providers Interviews with the most knowledgeable informants Recording	Semi-structured interview Use and analysis of secondary data/Triangulation of a variety of data sources Qualitative and quantitative data combined Pattern matching within and across cases/Search for the “why” behind relationships Comparison with literature Discussion within research team
**Construct validity** (whether a study investigated what it claimed to investigate using appropriate operational measures)	Adoption of questions and measures from previous research in the field of SME resilience in general and in a wider sense Linkage of protocol to a theoretical model	N/A	Multiple sources of information (e.g. different people per company, people from other industries, documents from the firms, direct observation) Independent interviews by several researchers	Iterative tabulation of evidence for each construct Review of case protocol by interviewed people
**External validity** (whether the findings from the specific cases are applicable elsewhere)	Identification of appropriate theoretical research questions with possible a priori constructs	Focus on a specified population with theoretical, not random, sampling: Deep and profound analysis of the case company’s context, industry, strategy and overall situation and clear description of all cases selected	Analysis of Interviews, Documents, Media on the case context Interviews with experts from the field of SME collaboration and resilience to challenge and broaden the data	Review of case protocol and interpretations by experts from the SME and organizational resilience field Analytical generalization on data patterns emerging

**Table 4:** Overview of Interview Results

Company	A	B	C	D
Role/Ownership	Crucial supplier 100% family owned	OEM 100% family owned	OEM Private equity	Crucial supplier 40% family owned + cross-investments
*Customers:* Type of Customers	*Retailers, wholesalers*	*Investment groups, public tenders, starting with retailers*	*Public authorities*	*Large companies*
*Contractual Investments*				
Product Flow	Depending on customer: standardized and large lot sizes (focus)	Individualized products for each customer	Individualized products for each customer	Product of agreed quality delivered in negotiated lot sizes
Financial Flow	Standardized payment process, prepayment expected from customers until certain relationship is established	Standardized payment process	Individually negotiated payment processes and terms	Payment process as negotiated
Information Flow	Continuous and timely support with hotline and individual personal contact standard information flows about product	Sales force and contractually agreed training (execution is categorized as relational instead, see below)	Timely information on production and delivery large sales department (execution is categorized as relational instead, see below)	Standard information flows
*Relational Investments*				
Product Flow	Depending on customer: highly individualized lot sizes	Regular events and personalized support	Personalized support and solutions to quality problems, including long warranty period	None
Financial Flow	No prepayment mandatory after a certain period of partnership	Silent passive SCF usage to secure payments of customers	SCF for international customers	Trade credit used often and late-payment opportunities offered to customers
Information Flow	Advice on product individualization regular visits by worldwide sales force individualized support from sales team: “We enable our customers to be creative and focus on their business, we ensure the rest.” No contracts with majority of partners	Large sales department for personal contact exchange of political and industry-related information individualized purchase advice for customers centre for training product users	Large sales department for personal contact exchange of political and industry-related information	Joint innovation in rare cases
*Suppliers:* Type of Suppliers	*One essential supplier group that consists of many companies; alternatives available worldwide*	*4 or 5 strategic partners,* including backups	*80% inter-company* *20% chemical companies*	*Suppliers of crucial ingredients*
Contractual Investments				
Product Flow	Raw materials delivered via standardized process for basic ingredients but in defined lot sizes and of agreed quality in special cases	Raw materials delivered after long negotiation process resulting in framework contracts with the most important suppliers from Asia; defined lot sizes and agreed quality	Mainly standard ingredients with some highly individualized and rare raw materials	Rare materials based on standard processes due to limited bargaining power
Financial Flow	Standard payment process after delivery	Payment process as negotiated	Prices defined on financial markets, limited room for negotiation, and focus on standardized processes hedging	Standardized processes
Information Flow	Standard delivery information	Personalized delivery information (execution is categorized as relational instead, see below)	Information on quality standard delivery information	Standardized information exchange
*Relational Investments*				
Product Flow	None	None	None	None
Financial Flow	Extracontractual prepayment options in some cases	Highly reliable partner in negotiations (especially financial ones) to differentiate company from other customers partial prepayment common despite absence of contractual agreement additional refund systems	–	Cross-investment with partners
Information Flow	Organization of events and competitions for suppliers at the company headquarters regular visits from the CEO, his team, and a worldwide salesforce investments into branding and team spirit regular information exchange on quality of raw materials to be delivered (often agricultural products that depend on climate) on personal basis (sales force); regular exchanges about industrial and political circumstances of each country	Regular personal visits from CEO and direct reports on some days, not only to negotiate but also to socialize informal exchange of information (about markets and government regulations) at personal meetings joint R&D/resource use coaching/training personalized delivery information via personal contact at purchasing department	Regular visits from purchasing team, including the CPO, with a focus on personal relationships use of joint R&D to individualize product with defined scope	Some basic joint R&D basic personal contacts for increased market and industry knowledge exchange
Role/Ownership	OEM Private Equity	OEM 100% family owned, not managed by owner	OEM 100% family owned, not managed by owner	Third-party logistics provider for several industries
*Customers:* Type of Customers	*Retailers, wholesalers*	*Large companies, smaller retailers*	*Retailers,* wholesalers	*Large variety of customers* from different industries and of different sizes
Contractual Investments				
Product Flow	Standard product, although some individualization possible delivered in defined lot sizes and at defined times to particular partners	Standard and individualized products that depend on customer contracts often provide for highly individualized delivery time, lot size, and product quality *(can also be categorized as a relational investment, see below)*	Highly individualized small-to-large lot sizes *(can also be categorized as a relational investment, see below)*	Highly individualized service negotiated individually, including individualized IT support *(can also be categorized as a relational investment, see below)*
Financial Flow	Standard payment processes (SCF used actively)	Standard payment process for majority of customers	Individually negotiated processes *(can also be categorized as a relational investment, see below)*	Individually negotiated processes *(can also be categorized as a relational investment, see below)*
Information Flow	Standard exchange of information about production and delivery	Standard exchange of information about production and delivery standard customer support	Standard exchange of information about production and delivery	Highly individualized information flows, often with own IT processes introduced *(categorized as relational investment despite being contractually agreed)*
*Relational Investments*				
Product Flow	–	Highly individualized contracts and products	Highly individualized small-to-large lot sizes	Highly individualized service negotiated separately, including individualized IT support
Financial Flow	–	Some individually negotiated payment terms (SCF used actively)	Individually negotiated processes	Individually negotiated processes
Information Flow	–	Some individual contact with largest customers sales force and personal contacts for customer care	Highly individualized information on production and delivery as well as additional product information regular visits from regional sales force (worldwide sales subsidiaries in place) and support in using the product exchange of informal information on market and customer base investment in dense networks within industry and politics	Highly individualized information flows, often with own IT processes in place regular visits from CEO exchange of informal information on market and customer base, offers of unexpected information (“to know the end customer better than the customer”) open communication about failures speaking the same language being reliable with deadlines and issue to be resolved
*Suppliers:* Type of Suppliers	**Crucial parts** (competitors)	**Basic material**	**High variety and many backups**	**Basic suppliers only** (transportation and machinery)
*Contractual Investments*				
Product Flow	Suppliers are also largest competitors, therefore only standardized products possible	Suppliers used only for basic products, but product quality and details usually negotiated individually	Basic products that can be sourced from various suppliers	Basic products that can be sourced from various suppliers
Financial Flow	Standard payment processes	Individually negotiated payment terms	Standard payment process	Standard payment process
Information Flow	Standard exchange of information about production and delivery	Standard exchange of information about product flow	Standard exchange of information about production and delivery	Standard exchange of information about production and delivery some audits for quality assurance (fully contractual)
*Relational Investments*				
Product Flow	–	–	–	–
Financial Flow	–	–	Being a reliable partner in payment always pay slightly early	–
Information Flow	–	Rare personal visits and contacts informal exchange of information about industry and market	Regular visits and audits from purchasing force to ensure quality being a reliable partner exchanges of information about industry and politics	–
